# Increase of Faecal Tryptic Activity Relates to Changes in the Intestinal Microbiome: Analysis of Crohn's Disease with a Multidisciplinary Platform

**DOI:** 10.1371/journal.pone.0066074

**Published:** 2013-06-20

**Authors:** Tore Midtvedt, Eugene Zabarovsky, Elisabeth Norin, Johan Bark, Rinat Gizatullin, Vladimir Kashuba, Olle Ljungqvist, Veronika Zabarovska, Roland Möllby, Ingemar Ernberg

**Affiliations:** 1 Department of Microbiology, Tumor and Cell Biology, Karolinska Institutet, Stockholm, Sweden; 2 Department of Surgery, Örebro University Hospital, Örebro, Sweden; 3 Inflammatory Bowel Disease (IBD) Unit, Stockholm Gastro Center, Sophiahemmet Hospital, Stockholm, Sweden; Karolinska Institutet, Sweden

## Abstract

**Objective:**

To investigate—by molecular, classical and functional methods—the microbiota in biopsies and faeces from patients with active Crohn's disease (CD) and controls.

**Design:**

The microbiota in biopsies was investigated utilizing a novel molecular method and classical cultivation technology. Faecal samples were investigated by classical technology and four functional methods, reflecting alterations in short chain fatty acids pattern, conversion of cholesterol and bilirubin and inactivation of trypsin.

**Results:**

By molecular methods we found more than 92% similarity in the microbiota on the biopsies from the two groups. However, 4.6% of microbes found in controls were lacking in CD patients. Furthermore, NotI representation libraries demonstrate two different clusters representing CD patients and controls, respectively. Utilizing conventional technology, Bacteroides (alt. Parabacteroides) was less frequently detected in the biopsies from CD patients than from controls. A similar reduction in the number of Bacteroides was found in faecal samples. Bacteroides is the only group of bacteria known to be able to inactivate pancreatic trypsin. Faecal tryptic activity was high in CD patients, and inversely correlated to the levels of Bacteroides.

**Conclusions:**

CD patients have compositional and functional alterations in their intestinal microbiota, in line with the global description hypothesis rather than the candidate microorganism theory. The most striking functional difference was high amount of faecal tryptic activity in CD patients, inversely correlated to the levels of *Bacteroides* in faeces.

## Introduction

Over the years, two major strategies have been proposed to define the possible role of microorganisms in Crohn's disease [1]; often named as “the candidate microorganism strategy” [2] and “the global description strategy” [3]. In spite of numerous attempts, it has so far been impossible to implicate a single microbial species or a group of specific microorganisms as the cause of the development of either CD or UC. Thus, the “candidate microorganism strategy” is presently very little focused on.

On the contrary, more attention has been put on the “the global description strategy”. In the beginning of the 1980s, the results of some investigations demonstrated decreased inactivation of intestinal tryptic activity [4], [5], and reduced levels or absence of trypsin-degrading microbes was hypothesized [6]. Since then, increasing amounts of data have been presented indicating that alterations in composition and function of the intestinal microbiota (IM), together with impaired epithelial barrier functions, partly governed by genetic and external factors, are involved in the pathogenesis and may be also in the aetiology of IBD, especially CD [7], [8], [9].

Methodological improvements in molecular, classical, and functional microbiology have given increased possibilities to investigate similarities and differences in IM on an individual as well as a group level [10]. Viewing IM from three different methodological angles may unmask even minor alterations in composition and function of IM in CD patients as compared with controls.

The aim of the present pilot study was to explore possible changes in the IM composition and function associated with CD. Utilizing a unique molecular method [11], a set-up of biopsies was investigated for presence of microbes. A parallel set-up of biopsies from the large intestine as well as faecal samples were cultivated on several different media [12], and by applying the MAC concept (MAC = Microflora Associated Characteristics) [13] four major microbial functions were studied in faecal samples. The short chain fatty acid (SCFA) pattern reflects a complex interplay between the host and its IM. Conversion of cholesterol to coprostanol and of bilirubin to urobilin reflects two important interactions on products undergoing an enterohepatic circulation and faecal tryptic activity (FTA) reflects the net sum of complex interactions between pancreatic derived trypsinogen and microbial/diet/host-derived activators and inactivators.

## Materials and Methods

### Selection of patients and controls

In this study four patients and five controls were entered, Table 1. They were all adults although the age range differed somewhat between the two groups. Patients with known CD and with active inflammation at the time of colonoscopy were chosen. None of the individuals had received antibiotics for two months before the time of sampling. For localisation of activity, see Table 1. Controls were selected from patients undergoing colonoscopy screening for cancer or polyps and where the investigation finding proved to be normal. None of these controls had any sign of inflammation or other disease macroscopically.

**Table 1:** Characterisation of patients and controls.

Age	Gender	Designation	Diagnosis/Reason for coloscopy	Active segments^b^	Previous GI surgery	Antibiotics last two months	Relevant medication
Patients^a^							
73	F	C1	Crohns disease (CD)	E	Appendectomy	no	no
31	F	C2	CD	BCDE	no	no	no
54	F	C3	CD	BCDE	no	no	no
25	M	C4	CD	BE	no	no	no
Controls^a^							
79	M	H1	screening		Appendectomy	no	no
54	M	H2	screening		no	no	no
74	F	H3	abdominal pain		no	no	no
78	F	H4	distention		no	no	no
34	F	H5	distention		no	no	no

a Patients: 25% male gender, mean age 46 years (25–73).

Controls: 40% male gender, mean age 64 years (34–79).

b B; colon ascendens, C; colon transversum, D; colon descendens, E; colon sigmoideum/rectum.

Originally a cohort of 17 IBD- and 16 non-IBD patients had been sampled among patients entered for endoscopy, and they were blinded to the investigators. For this study we realized that such a material would still be quite heterogenous. Therefore the code was broken and only patients suitable for analysis with active Crohn were selected, although the samples from these patients selected were still kept blind to those doing the analysis. Among the controls patients with divertculae/divertuculitis and irritated bowel syndrome (IBS) were excluded. Also recent antibiotics usage was considered. After this only 4+5 patients were suitable for analysis.

Before arriving at the endoscopy unit, all individuals completed a questionnaire regarding bowel habits, diet, concomitant medication, previous gastrointestinal surgery, use of probiotics or antibiotics and subjective food intolerances, previous diagnoses regarding the GI tract, presence of other diseases or known allergies (see Tables S1 and S2). As part of the preparation for the colonoscopy, the patients were informed to avoid a diet rich in fibre and raw vegetables/fruits with peels during the week preceding the colonoscopy. On the day before the procedure only clear liquids were allowed. The laxative used for pre-exam bowel cleansing was sodium phosphate (Phosphoral®/Fleet enema, Ferring Pharmaceuticals, Denmark) at a total volume of 90 ml divided into two doses with four hours interval. The patients underwent colonoscopy using a standard Olympus GIF endoscope (Olympus Europe GmbH, Germany) under mild benzodiazepine sedation with midazolam (Dormicum, Roche, Sweden) and/or intravenous administration of Alfentanil (Rapifen®, Janssen-Cilag, Sweden).

The study protocol was reviewed and approved by the local ethical committee, Research Ethics Committee South, Karolinska University Hospital South, SE 141 86 Stockholm (permit 362/02), Sweden. Patients and controls were recruited from entries to the endoscopy department at Ersta Hospital, Stockholm, Sweden. All signed a written consent form before participation.

### Sampling

Both in patients and controls, biopsies were sampled with standard biopsy forceps in the middle part of the transverse colon, Table 1. Two parallel biopsies were taken and were put into separate test tubes containing a small volume of sterile reducing buffer (Isotonic phosphate buffered solution (PBS) and thioglycollate, 1 g/L) at +4°C. All endoscopies were uneventful and no complications were observed. Faecal samples were collected before taking any laxative. All samples were coded and frozen at −70°C and later analyzed in a blind fashion.

### DNA isolation, probe and sample preparation

DNA was isolated from each biopsy using QIAamp DNA Stool Mini Kit (Cat No: 51504, Qiagen Nordic, Sweden). This DNA was subsequently amplified using Whole Genome Amplification kit (WGA) with Phi29 polymerase according to the manufacturers' instructions (GenomiPhi DNA Amplification Kit, Cat. No. 25-6600-01, GE Healthcare). The resulting amount of DNA was usually 1–3 microgram. All enzymes were from New England Biolabs (USA).

The DNA from the biopsies was further processed for microarray hybridization by generating “NotI representation” (NR) libraries. The NR probes were prepared as described earlier [11], [14], [15]. In brief, this involved DNA digestion with NotI restriction enzyme, ligation to NotI-linkers, digestion with Sau3A restriction enzyme, immobilization on Dynabeads M-280 Streptavidin “Dynal” and finally washing and ligation to magnetic beads with Sau3A-linkers. The enriched DNA was amplified by PCR using universal and linker-primers. PCR conditions were the following: 2 min at 95°C, then 35 cycles of denaturation (45 sec at 95°C), annealing (40 sec at 64°C) and synthesis (2 min 20 sec at 72°C). Thereafter, 200–400 ng of NR was labelled by PCR as described above but in the presence of 1,25 nM of Cy5-dCTP (or Cy3-dCTP).

### Microarray hybridization and bioinformatic analysis

Slides were printed with DNA probes (oligonucleotides) as described earlier [15] using QArrayMini (Genetix, England) according to the manufacturer's instructions. Altogether 90 bacterial specific oligonucleotides were synthesized by Invitrogen Ltd (UK). For this purpose they were selected from publically available information in data bases. Six different DNA preparations were used as controls: total human DNA, salmon sperm DNA, plasmid DNA pBluescript II KS, lambda DNA, NotI linking clone NL1-024 and *E. coli* K12. Each specific DNA probe was spotted on microarray slides in six repeats to comply with variation and reproducibility. The biopsy derived NR libraries were hybridized to these microarray slides using a TECAN HS400 (Tecan Trading AG, Switzerland) according to the manufacturer's protocols. The microarray slides were scanned in GenePix 4000B after hybridization and the results were analyzed using GenePix Pro (4.0) program (Amersham Biosciences Europe, Sweden), which provides an overview summary of intensity of hybridization on each spot, and calculates the mean hybridization signal for each spot. Clustering analysis was based on simple Euclidean distances.

### Colony hybridization and identification of microorganisms

NotI representations from four CD patients and five controls were cloned in the pUC18 plasmid and introduced in to *E. coli* strain XL-1 Blue from Stratagene to create full coverage libraries (USA) as described earlier [11]. Each library was plated on 5 LB-agar plates to a 1000 colony density. Colony hybridization was performed according to standard protocols. DNA probes were labelled with ^32^PdCTP by the random oligonucleotide priming method. For sequence validation and identification of specific microorganisms PCR was performed with the probes summarized in Table 2.

**Table 2:** Oligonucleotides used for preparation of probes.

Name	sequence	bacteria	used
bact3 F	GAT CTT TGC GGA TTG TTC CAG	Bacteroides fragilis clone 38-F	for PCR analysis
bact3 R	CGC CCA AAA ATT AGA CGA GTA	Bacteroides fragilis clone 38-F	for PCR analysis
mus1 F	GCC GCC GTG GAG AGC ATG AC	unknown bacteria	for PCR analysis
mus1 R	CCG CTC GCT CTG CCC TGG CTA	unknown bacteria	for PCR analysis
37un F	GCCGACGTTGAACATTACCAAG	unknown bacteria	for PCR analysis
37un R	ATGGCGTTGTTGGATATGCTCT	unknown bacteria	for PCR analysis
gr1 F	GGT CTG TGA CAG GCT CAA CGG	Uncultured bacterium clone HA0AAA13ZF	for PCR analysis
gr1 R	GCT CGG AAT ACA TCT TTA TGG G	Uncultured bacterium clone HA0AAA13ZF	for PCR analysis
gr3 F (14un)	CGG CGC TTG GAT TTA GTA TGT C	unknown bacteria	for PCR analysis
gr3 R (14un)	CCA TTT GCT TAC CGC CAT ATC T	unknown bacteria	for PCR analysis
gr4 F	GAG AAA TAG AAT CTT CGG AAC A	unknown bacteria	for PCR analysis
gr4 R	ACA TAA TAA GGC AAA GTG TAA CC	unknown bacteria	for PCR analysis
gr5 F	GGC CTT AAC GCT AAT CTA CTG C	unknown bacteria	for PCR analysis
gr5 R	GGC CTG CTC CTC GGT AAA CTC	unknown bacteria	for PCR analysis
vpi F	GAA CCA GAG AGC CGA CAG TCT	Bacteroides thetaiotaomicron VPI-5482	for PCR analysis
vpi R	ACA ATG GCC TCT ATC GTG TTT	Bacteroides thetaiotaomicron VPI-5482	for PCR analysis
NotAntBio	Biotin-CAGCACTGACCCTTTTGGGACCGC		for NotI representaition
NotAntComp	GGCCGCGGTCCCAAAAGGGTCAGTGCTG		for NotI representaition
SauZgtBlock	GATCCTCAAACGCGT-block		for NotI representaition
SauZgtComp	GGCGATCTATCCTAGAGCCCGTACGCGTTTGAG		for NotI representaition
Anti-univ	CAGCACTGACCCTTTTGGGACC		for NotI representaition
Zgt99	GGCGATCTATCCTAGAGCCCGT		for NotI representaition

For identification of bacteria common to CD patients and controls, and unique to each of these respectively, a reference pool was made of NotI libraries from three CD patients and from three controls. These two pools were then cross hybridized against each other, which allowed identification of common species as well as unique bacterial species in CD patients and controls, respectively.

### Cultivation methods

The biopsies were thawed, mashed and suspended in 1 mL sterile PBS, and cultivated on selective and non-selective media by spreading out 0.1 mL of five 10-step dilutions on appropriate agar plates (Table 3). Bacterial colonies were counted by visual inspection after one to five days of incubation at 37°C, aerobically and anaerobically. Faecal samples were thawed, suspended and diluted in PBS and further treated as the biopsies.

**Table 3:** Isolation and identification of bacterial colonies from biopsies and faeces.

Group of bacteria	Agar plate used	Aerobic/anaerobic inoculation	Identification methods
*E. coli*	CLED^1^	aerobic	Colony appearance, biochemical tests^2^
Alpha-streptococci	Blood^3^	aerobic	Colony appearance, green haemolysis
Other aerobic *Streptococcus spp.*	Blood^3^	aerobic	Colony appearance Gram staining,
*Staphylococcus spp.*	Blood^3^	aerobic	Gram staining, haemolysis
Other facultative aerobic bacteria	Blood^3^	aerobic	Gram staining
*Lactobacillus spp.*	Rogosa^4^	anaerobic 3–5 days	Colony appearance, Gram staining
Bifidobacteria	Bifido^5^	anaerobic 5 days+recultiv. aer+anaer	Gram staining
*Bacteroides spp.*	BKV^5^ (Blood-kanamycin vancomycin)	anaerobic 5 days+recultiv. aer+anaer	Gram staining

1 CLED, Difco labs, Detroit, USA.

2 Indol test+, lysine decarboxylase+ (and lactose+ in the agar plate).

3 Blood, Oxoid Ltd, UK.

4 Rogosa, De Man, Rogosa and Sharp agar, Merck, Germany.

5 Karolinska Hospital, Sweden.

After incubation over 2–5 nights, the different groups of bacteria were identified according to standard methods (Table 3) [12]. The number of bacteria per sample were calculated as colony forming units (cfu∶s) per biopsy or per g of faeces. The size of each biopsy varied at most threefold, but the amount of faeces to be analysed was measured. Due to logistic reasons a faecal sample from one of the CD patients was not available for analysis.

### Metabolic parameters

At analysis, the thawed faecal samples were homogenized and thereafter handled as described in details elsewhere [16]. SCFAs were determined with gas-liquid chromatography (GLC) utilizing 2-methylbutyric acid as internal standard, and shown as mmol/kg of faeces [17], [18]. Determination of cholesterol and coprostanol was performed by GLC, and the data were expressed as percent coprastanol out of the total amount of cholesterol and coprostanol present [19]. Urobilin was determined spectrophotometrically and phenolsulfonphtalein was used as an artificial standard for urobilinogen-aldehyd. The spectrophotometric values were converted to mmol urobilinogen/kg faeces using 596 D as the molecular weight of urobilinogen [20]. Faecal tryptic activity (FTA) was determined spectrophotometrically utilizing N-benzoyl-DL-arginine-paranitroanilide HCl as substrate. Bovine pancreatic trypsin (Type III, Sigma, USA) was used to construct a standard curve and the enzymatic activity was expressed as mg active trypsin/kg faeces [21].

### Statistics

Student's t-test was used for analysis of short chain fatty acids, Mann-Whitney's nonparametric test for comparison of MAC-levels and Pearsons correlation test for probing the relationship between Bacteroides and FTA, all calculated by Graphpad Instat ver. 3.06 (Graphpad Software Inc, USA).

## Results

### Molecular characterisation and comparisons

**Figure 1:**
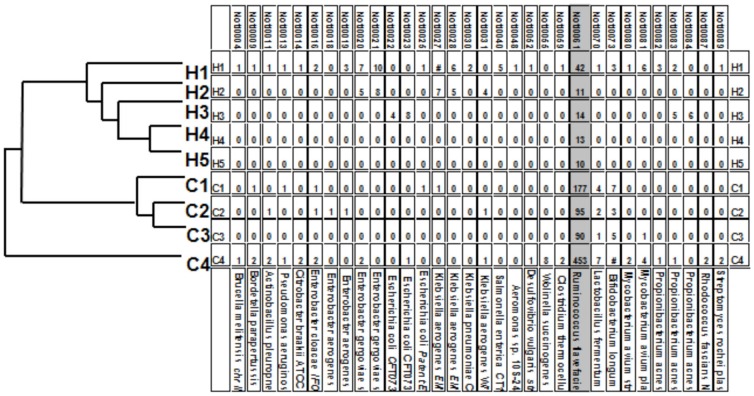
Hybridization of control and CD patients “NotI representation” libraries to oligonucleotide-probes in biopsies. Numbers reflect strength of signal after hybridization. Corresponding bacterial names are shown in the bottom row. *Ruminococcus flavefaciens* demonstrating the largest difference in the signal strength is shown in grey. The signals were clustered into a dendrogram using Euclidean distances depicted to the left. Crohn patients (C1–C4); controls (H1–H5).

The biopsy generated NR-libraries were hybridized against the 90 bacterial oligonucleotides derived from publicly available databases. The specificity was validated by sequencing the DNA from four hybridizing spots, and we found DNA sequence specific for the expected bacterial strain in each case. Human sequences represented 10–40% of sequences present in NRs. Using Euclidean Distance two clusters were revealed: one with only CD patients and one with the controls (Figure 1). However, it was obvious that this approach to match the sample microbes to pre-selected sequences from data-bases of known DNA-sequences of commensal microbes provided limited information, when applied to the microflora with very large heterogeneity and many unknowns. Many such probes did not provide any signal. We therefore exploited a technological platform developed by us which allows identification of unknowns and also of non-abundant species.

### Differential hybridization using CD patients and a pool of control individuals

**Figure 2:**
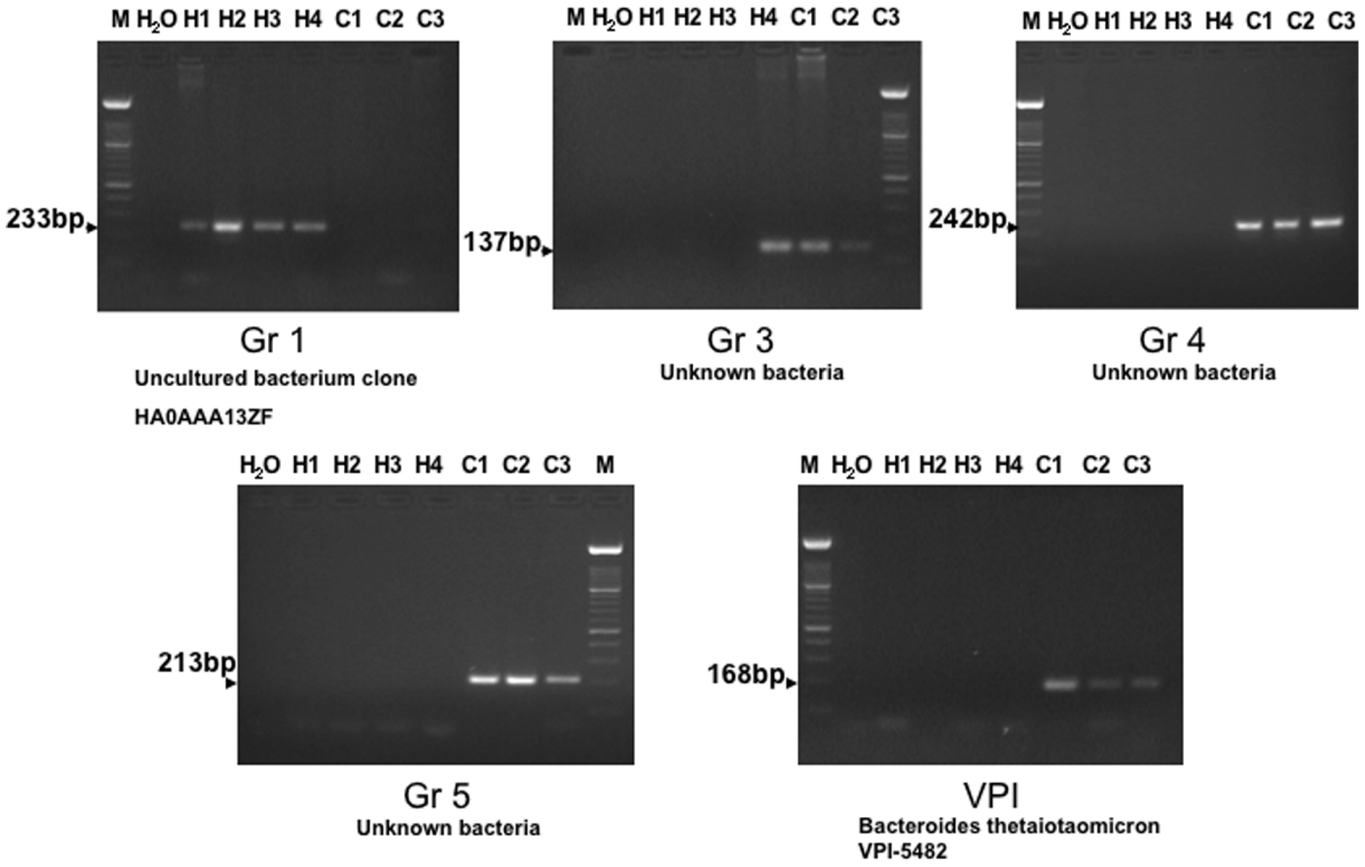
Bacterial DNA unique to Crohn patients (C) or controls (H) as demonstrated by PCR-amplification using primers according to table 2. PCR group 1 bacterial DNA was found in all four controls, while groups 3–5 and VPI were only detected in biopsy samples from the Crohn patients. By sequencing of the PCR-products (not shown) it could be shown that groups 1–5 represent bacteria not previously identified, while VPI represents *Bacteroides thetaiotaomicron* VPI-5482.

Based on libraries generated from the reference pools of control and CD patient samples, analyzis of hybridization patterns revealed that 92,6% of the bacteria were the same in CD and control pools. However, 2,8% of the bacterial colonies containing cloned sequences were specific for CD patient samples and 4,6% were unique to the control samples. A number of such colonies were isolated and further characterized by PCR using primers shown in table 2 (Figure 2). After sequencing of five such bacterial DNAs we could show that only one was previously undentified (data not shown).

### Cultivation of bacteria

**Figure 3:**
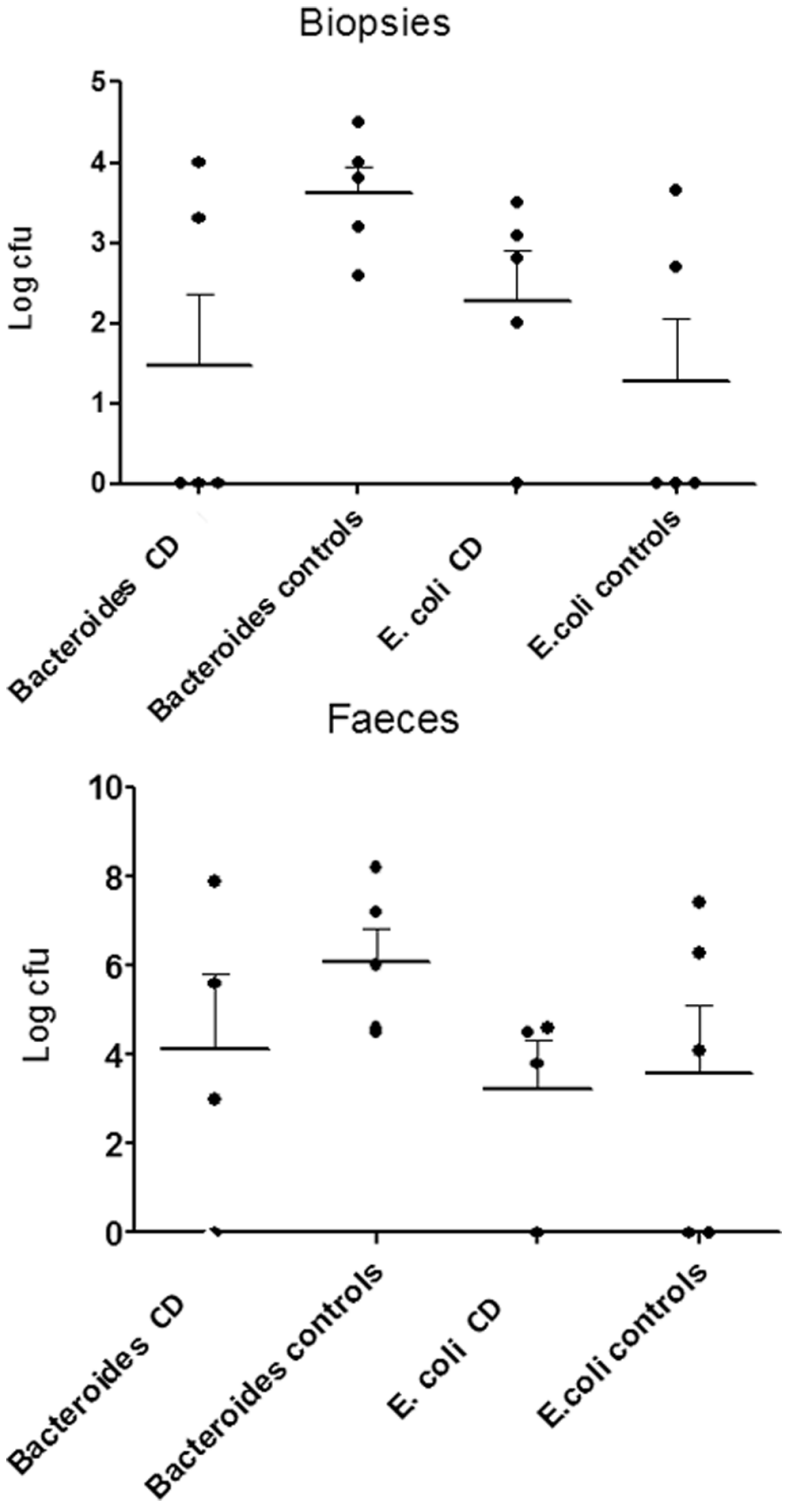
Cfu∶s of *Bacteroides spp.* and of *E.* *coli* per biopsy or per gram faeces. CD patients versus controls. Horisontal line depicts mean number of log cfu∶s ( = geometric mean of cfu∶s) and the upper T depicts SEM. Note that three out of five biopsies from CD patients did not show any growth of Bacteroides, while all control biopsies did (p = 0.054, Mann-Whitney).

For seven of the eight bacterial groups analyzed (Table 3), lower numbers of bacteria were observed on biopsies obtained from CD patients than from healthy controls, and the geometric mean of the total number of bacteria detected was more than five times lower on both biopsies and in faeces from CD patients compared to controls (data not shown). Figure 3 shows the distribution of *Bacteroides spp.* (now also referred to as *Parabacteroides*) and *E. coli* in biopsies and faeces. The most striking difference was seen for *Bacteroides spp.* which was detected on only 2 out of 5 biopsies from CD patients compared to all five from the controls (p = 0.054). Coliforms, mainly *E.coli*, was the only group being more commonly found on biopsies from CD patients than from the controls (four vs. two, respectively, out of five; Figure 3).

Also in the faecal samples, Bacteroides were less frequent in faecal samples from CD patients than from healthy controls, whereas the amounts of coliforms were similar. In general, the CD patients exhibited a slightly less diverse IM than the controls.

### Metabolic parameters

**Figure 4:**
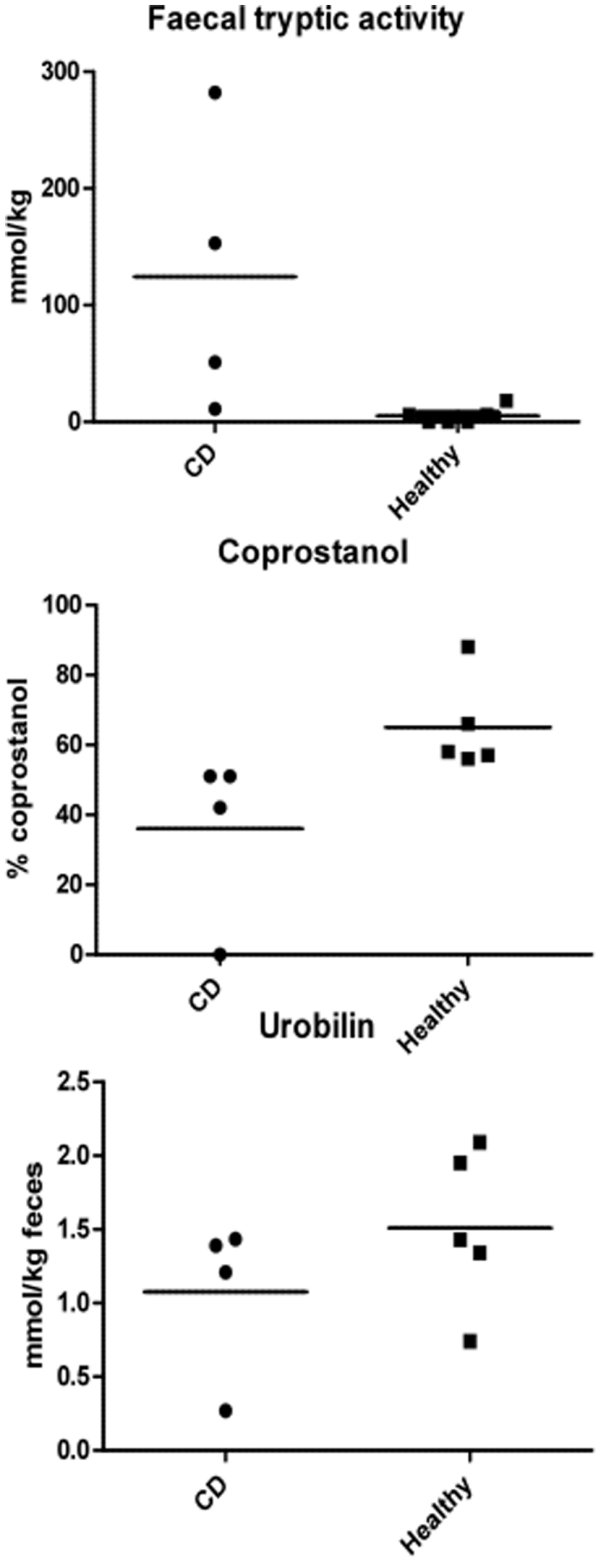
Levels of three MAC∶s in faecal samples from Crohn patients versus healthy controls. Horizontal line depicts mean value.

**Figure 5:**
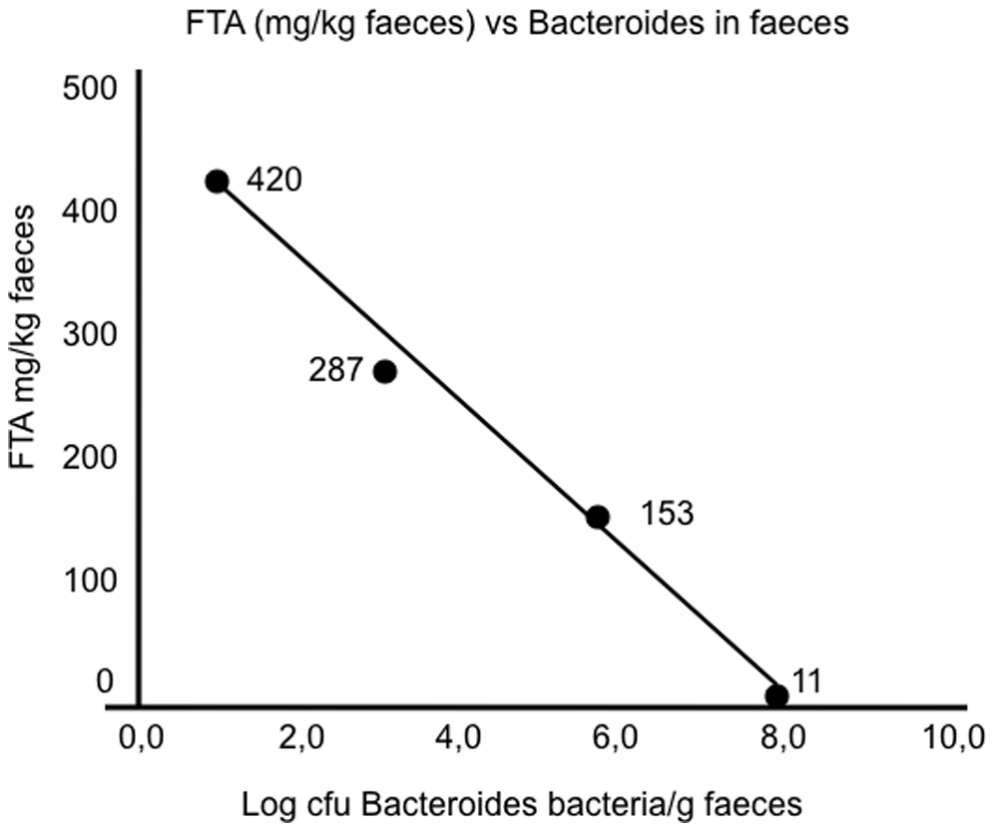
Relation between log nr of Bacteroides in faeces and faecal tryptic activity (FTA). Numbers denote FTA activity, p-value = 0.0011 (Person). It is to be noted that all control samples had high numbers of Bacteroides and low levels of FTA (Figures 3 and 4). *denotes a CD patient with ileo-caecal resection, why he was not included in other data in this manuscript. One CD patient was not included since his faecal sample was not subjected to cultivation for technical reasons.

As shown in table 4, no significant differences were seen between the mean values of faecal propionic, i-butyric and butyric acids, caprioic or valeric acids, nor in the total amounts of SCFAs (Table 4). However, the total amounts of the valeric (C_5_) and caproic (C_6_) forms tended to be lower in the CD patients as compared to controls. Furthermore, the CD patients showed a significantly lower conversion rate of cholesterol to coprostanol (p = 0.02) as compared to the controls and a similar pattern was seen when investigating the levels of urobilin (Figure 4). On the contrary, the levels of FTA were significantly higher in CD patients than in controls (p = 0.03). Interestingly, the levels of FTA were inversely correlated to the numbers of Bacteroides in the faecal samples (Figure 5).

**Table 4:** Microflora associated characteristics (MACs) of the CD patients (n = 4) and controls (n = 5). Concentrations of relevant short chain fatty acids (SCFAs) in faeces (mmol/kg) (mean and range).

	Acetic acid	Propionic acid	i-Butyric acid (i-C_4_)	i-Valeric acid (i-C_5_)	i-Caproic acid (i-C_6_)	n-Butyric acid (C_4_)	n-Valeric acid (C_5_)	n-Caproic acid (C_6_)	Total SCFAs
Crohn patients	94.3(79–106)	12.5(10–14)	2.1(1.5–3.1)	2.6(1.5–4.1)	0.2(n = 2)	28.4(9–57)	2.5(0.7–3.7)	0.9(0.1–3.2)	147(121–187)
Control patients	91.8(40–162)	20.9(6.2–48)	3.3(1.9–5.6)	4,4(2.7–8)	0.2(0–0.3)	18.2(9.3–33,6)	4.5(2.2–7)	2.6(0.2–9.3)	145(63–262)

## Discussion

The heterogeneity of CD patients' pathophysiological conditions, as demonstrated in Table 1, reflects the everyday clinical situation. We decided – in this study – to take a closer look on biopsies from one location only, i.e. from colon transversum. Differences in mucosal microbiota between the two groups might then incite to more basic alteration than if biopsies taken from only active immunological sites had been investigated. As mentioned earlier, the main intention behind this study was to view the IM from three different methodological angles in order to unmask even minor alterations in composition and functions.

The application of culture-independent techniques based on molecular methods, introduced in microbiology more than two decades ago, has overcome shortcomings of conventional cultivation methods. Over the years, many different methods and modifications of methods have been applied and most of them have been based on amplification of the 16S rRNA gene. Taken together, studies utilizing these methods have provided us with important information about the composition – and complexity – of the intestinal flora. However, intrinsic disadvantages of these approaches limit their application. When utilizing 16S rRNA genes, the problem is that this gene is highly conserved and therefore the same sequenced fragment sometimes can represent different species. Moreover, different fragments may also represent the same species.

The main idea of our approach was not to sequence complete genomes or study all genes. We assigned special signatures for particular microorganisms/genes and analyzed these signatures in the colonic samples. Among them we selected known or most interesting microbial species/strains found at least once in the human gut and designed 90 oligonucleotides (55 bb long) for them. Molecular chips with 550 nucleotide dots were constructed and NR probes from both the CD patients and controls were prepared. The main conclusions from these experiments were that the microarray hybridization was specific, but that the selection of representative probes pose a problem with such a broad heterogeneity of microbes and many previous unknowns as in the gut microbiome.

With our alternative approach these shortcomings can be taken care of. The Not I library approach offer several advantages. It allows detection of relatively rare species when combined with a subtraction approach as here. It is insensitive to whether the DNA sequence has been detected before, i.e. formerly unidentified microbial sequences are treated similar as well-knowns. It is highly specific and can also recognize strain variations, in contrast to 16S RNA. It allows cohort-wise comparisons, e.g. all patients versus all controls, allowing us to identify cohort-specific bacterial sequences. In all these aspects the Not I-library approach must be considered as a complement to sequencing of 16S RNA, and in fact for many purposes advantageous to that widely used method. The only drawback is that it is relatively work intensive and not yet highly automated like many HTP sequencing protocols.

With Not1 libraries generated from patients and controls themselves we identified bacteria present in CD patients only and also microbes unique for the control samples. Not surprisingly, we found most microorganisms present in both libraries. By hybridization analysis we found that 92.6% of microorganisms in both mixtures (i.e. from 10.000) are the same (or very similar) 2.8% are present only in CD patients and 4.6% only in the controls. We sequenced 5 such microorganisms unique to the one or the other group and confirmed that they were specific either to CD patients or controls. Among those 5 tested microorganisms only one was previously known. The most remarkable difference between the two cohorts was that the genus Bacteroides was present in all controls but not detected in 3 out of 4 CD patients. This will be commented upon later.

The reduced number of bacteria attached to the mucus of the biopsies might be due to a sum of factors, such as activated immune defence, increased cell turnover, altered physiochemical conditions as oxygen tension, red/ox potential etc. Partly, some of these same factors may also be involved in the lesser diversity found in faecal samples from CD patients [8]. The larger numbers of coliform bacteria, found on the CD patients' biopsies [22], [23], has been hypothesized to have a pathogenic role. Whether and to what extent these findings are of aethiopathological importance, need to be substantiated.

Taken together, the results regarding SCFAs, coprostanol and urobilin might indicate altered entero-metabolic functions in CD patients, both with regard to the findings in our controls as well as findings in large cohorts of healthy adults [16], [17], [18], [19], [20], [21], [22]. The most striking results in this study was the high values of FTA simultaneously with a low number of *Bacteroides* in the CD patients.

It might be relevant to briefly summarize how microbes are involved in FTA. Trypsinogen is secreted from the pancreatic glands into the small intestine were it is activated to trypsin. The importance of a bacterial intestinal breakdown of trypsin is clearly demonstrated in comparative studies of germfree and conventional animals, i.e. animals harbouring a normal intestinal flora. The latter always demonstrate zero values of FTA whereas the former animals always demonstrate high levels [23], [24]. To the best of our knowledge, man is the only mammalian species so far investigated in which FTA can be found in presumably healthy individuals [21], [25], [26]. The mechanism(s) or reason(s) for this human feature is (are) not known. Some few years ago, it was shown that a strain of Bacteroides was able to break down trypsin both *in vitro* and *in vivo* [27]. Our microbiological findings in this study strongly indicate less Bacteroides in CD patients than in the controls, both by molecular and by classical methods. Applying the global description strategy, the sum of our observations indicates that CD might be due to a reduced number or absence of some metabolically active microbes within the Bacteroides group. In fact, our observations fit very well with the theory suggesting that CD is caused by a “dysbiosis” of the intestinal flora [28].

Although we here have observed the status in established CD, it is of interest to speculate how trypsin, an important endogenous enzyme, might act as a triggering factor in CD? It has been shown in conventional mice that intra-colonic administration of trypsin cause mucosal damage [29] whereas high levels of intra-colonic trypsin in germfree animals never trigger any mucosal damages [4], [30], [31], [32], [33], [34]. Obviously, there is a need for “trigger” microbe(s) or mechanism(s). Whether and to what extent presence of *E. coli* in high numbers on biopsies, as found in some previous investigations, as well as in this study, constitute a triggering event needs to be further elucidated [21], [22]. Also in our study, a higher number of *E. coli* was seen in the patients, although specific virulence factors were not investigated.

Adopting the view recently presented by Man et al [35], it might be relevant to characterize the initial mechanisms in CD as an “out-in” event - a dysbiosis. After the mucosal line is broken, then a series of “in-out” events may take place. Results of some recent studies indicate that various proteases (trypsin, mast cell derived tryptase etc) are implicated in the pathogenesis via activation of specific cell-bound receptors (PARs) resulting in a long series of pro-inflammatory events [36], [37]. A reduced tissue production of pancreatic secreted trypsin inhibitor following an initial mucosal damage in CD patients [37] substantiates this theory.

### Concluding remarks

We have viewed the IM in CD patients utilizing three principally different methods. The most striking functional difference was high amounts of faecal tryptic activity in CD patients, inversely correlated to the levels of *Bacteroides* in faeces. Taken together, our results indicate that Crohn's disease patients have a compositional and functional dysbiosis in their intestinal microbiota. Employing a global description strategy should stimulate to increased efforts working out the complex microbe/host interplay going on in the GI tract of CD patients. A better understanding of this interplay may open up for new therapeutic interventions.

## Supporting Information

Table S1 Questionnaire to patients and controls.
(DOC)

Table S2 Patient characteristics according to questionnaire.
(DOC)
