# Supplementary material for: Increase of Faecal Tryptic Activity Relates to Changes in the Intestinal Microbiome: Analysis of Crohn's Disease with a Multidisciplinary Platform
Source: PLoS One. 2013 Jun 20;8(6):e66074. doi: 10.1371/journal.pone.0066074 (PMC3688706; doi:10.1371/journal.pone.0066074)
Supplement: Table S1 — Questionnaire to patients and controls. (DOC) [file pone.0066074.s001.doc]

**Table S 1. Questionnaire to patients and controls**

When was the last time you were treated with antibiotics?

Do you consume probiotics on a regular basis?

Number of defecations per week,quality of stools?

Have you had G-I surgery performed?

What medication are you on?

Have you been diagnosed with a G-I condition by a physician? What?

Have you spent a longer period abroad? Which countries?

Do you perceive any kind of food intolerance? If so, against what?

Do you have diabetes?

Do you consider yourself producing excessive amounts of flatus?

Are you often distended in your abdomen?

Do you suffer from abdominal pain/cramping? In any special situation?

Do you have any known allergies?
