# Supplementary material for: Increase of Faecal Tryptic Activity Relates to Changes in the Intestinal Microbiome: Analysis of Crohn's Disease with a Multidisciplinary Platform
Source: PLoS One. 2013 Jun 20;8(6):e66074. doi: 10.1371/journal.pone.0066074 (PMC3688706; doi:10.1371/journal.pone.0066074)
Supplement: Table S2 — Patient characteristics according to questionnaire. (DOC) [file pone.0066074.s002.doc]

**Suppl table 2A. Patient characteristics according to questionnaire. Diagnosis.**

| No | Birth year | Sex | Distention | Diabetes mellitus | Antibiotics  within last  two months | Diagnosis |
| --- | --- | --- | --- | --- | --- | --- |
| H1 | 24 | m | N | N | N | Polyps |
| H2 | 49 | m | Y | N | N | Healthy |
| H3 | 29 | f | Y | N | N | Healthy |
| H4 | 25 | f | Y | N | N | Abdomin pain |
| H5 | 69 | f | Y | N | N | Abdomin pain |
| C1 | 30 | f | N | N | N | CD active Il/colon |
| C2 | 73 | f | N | N | N | CD active Proctosigm |
| C3 | 50 | f | N | N | N | CD ileitis |
| C4 | 79 | m | N | N | N | CD colitis |

**Suppl table 2 B. Patient characteristics according to questionnaire.**

| No | Probiotics | Defecations | Surgery  Stomach-gut | Ongoing  Medication | Food allergy | Flatulence |
| --- | --- | --- | --- | --- | --- | --- |
| H1 | N | 1/d | Appendectomy 48 | Plen,betabl | N | N |
| H2 | N | 1-2/d | N | N | Spices | Y |
| H3 | N | 1/d | Hemorojds | Beta,plend,zocord,cozaar,tiazid | N | Y |
| H4 | N | 1/d | Anal fissure | N | Raw vegetables, fruit,ägg,sour milk,fermented bread | Y |
| H5 | N | 1/d | N | N | Fat, sweets, ice cream | Y |
| C1 | Y | 3/d | Appendectomy | Levaxin,sotal,plend,tromb,spirol,hipr,citalop, | N | Y |
| C2 | N | 1/d | N | Aerius,tryptiz | N | N |
| C3 | N | 1/d | N | Fluoxetin,zopiklon | N | Y |
| C4 | N | 2/d | N | Behep,brica,pulmic | Fat | N |
